# Insect herbivory increases from forest to alpine tundra in Arctic mountains

**DOI:** 10.1002/ece3.8537

**Published:** 2022-01-24

**Authors:** Elena L. Zvereva, Vitali Zverev, Mikhail V. Kozlov

**Affiliations:** ^1^ Department of Biology University of Turku Turku Finland

**Keywords:** elevational gradient, insect herbivory, open‐top chambers, specific leaf area, temperature, woody plants

## Abstract

Current theory holds that the intensity of biotic interactions decreases with increases in latitude and elevation; however, empirical data demonstrate great variation in the direction, strength, and shape of elevational changes in herbivory. The latitudinal position of mountains may be one important source of this variation, but the acute shortage of data from polar mountains hampers exploration of latitude effects on elevational changes in herbivory. Here, we reduce this knowledge gap by exploring six elevation gradients located in three Arctic mountain ranges to test the prediction that a decrease in herbivory occurs with increasing elevation from forest to alpine tundra. Across the 10 most abundant evergreen and deciduous woody plant species, relative losses of foliage to insect herbivores were 2.2‐fold greater at the highest elevations (alpine tundra) than in mid‐elevation birch woodlands or low‐elevation coniferous forests. Plant quality for herbivores (quantified by specific leaf area) significantly decreased with elevation across all studied species, indicating that bottom‐up factors were unlikely to shape the observed pattern in herbivory. An experiment with open‐top chambers established at different elevations showed that even a slight increase in ambient temperature enhances herbivory in Arctic mountains. Therefore, we suggest that the discovered increase in herbivory with elevation is explained by higher temperatures at the soil surface in open habitats above the tree line compared with forests at lower elevations. This explanation is supported by the significant difference in elevational changes in herbivory between low and tall plants: herbivory on low shrubs increased fourfold from forest to alpine sites, while herbivory on trees and tall shrubs did not change with elevation. We suggest that an increase in herbivory with an increase in elevation is typical for high‐latitude mountains, where inverse temperature gradients, especially at the soil surface, are common. Verification of this hypothesis requires further studies of elevational patterns in herbivory at high latitudes.

## Graphical abstract

Our study of insect herbivory along six elevational gradients located in three Arctic mountain ranges demonstrated that losses of plant foliage to insects increased with increase in elevation across 10 woody species. We suggest that this elevational pattern is typical for high‐latitude mountains, where inverse temperature gradients, especially at the soil surface, are common.

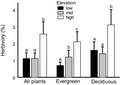

## 1 INTRODUCTION

Mountains harbor a major part of Earth's terrestrial biodiversity (Payne et al., 2020; Rahbek et al., 2019). Consequently, studies of biotic interactions in mountain regions are crucial for revealing the mechanisms that generate and maintain the high‐diversity mosaic of mountain communities in spatially and temporally variable climatic conditions. Sharp environmental gradients in mountains offer a powerful tool for elucidating the effects of abiotic factors on species diversity, trait evolution, biotic interactions, and ecosystem services at both the ecological and evolutionary time scales (Janzen, 1967; Körner, 2007; Rasmann et al., 2014; Schemske et al., 2009). With this respect, elevational gradients have some advantages over latitudinal gradients due to short spatial distances between localities with different climates and in similar day lengths at different elevations.

Current theory holds that the intensity of biotic interactions, and particularly of herbivory, is highest in warm and stable environments at low latitudes and elevations and that this intensity decreases toward high latitudes and elevations (Dobzhansky, 1950; Pellissier et al., 2012; Schemske et al., 2009). However, recent studies have revealed great variation not only in the strength and shape of both latitudinal and elevational changes in herbivory, but even in the direction of these changes as well (Moles et al., 2011; Moreira et al., 2018; Zvereva & Kozlov, 2021). Therefore, elucidating the factors that shape herbivory along environmental gradients is a formidable goal.

Various factors have been suggested to explain the variation observed between the outcomes of elevational studies of herbivory, including plant traits and climate (reviewed by Galmán et al., 2018). In particular, broad‐scale latitudinal differences in climatic variability, both diurnal and seasonal, may influence elevational gradients in biotic interactions because the rate of change in the biotic environment (e.g., in species composition), when moving from low to high elevation, is greater in tropical than in temperate regions (Ghalambor et al., 2006; Janzen, 1967). Moreover, the elevation of the alpine tree line decreases with increases in latitude (Körner, 1998). As a result, polar mountains show abrupt changes in their biotic environment (i.e., in the type of vegetation) at relatively low elevations.

Comparative studies of elevational changes in herbivory across latitudes are hindered by a geographic bias in research toward temperate mountains (accounting for 76% of the gradients reviewed by Moreira et al., 2018), whereas data from elevational gradients in high‐latitude (polar) mountains remain scarce (but see Kristensen et al., 2020). Filling in this gap in knowledge is especially important because ectothermic animals living at high latitudes are closer to their lower thermal limits (Deutsch et al., 2008) and may therefore show greater responses to the same absolute change in temperature compared to their counterparts living at optimum temperatures. Furthermore, polar regions differ considerably from temperate and tropical regions in terms of the rate of latitudinal changes in biotic interactions (Zvereva & Kozlov, 2021) and high‐latitude areas suffer most from the environmental changes due to exceptionally rapid climate warming (IPCC, 2018).

Elevational changes in herbivory may differ considerably among plant species, and these differences are partly related to their growth form (woody vs. herbaceous) and life history traits (evergreen vs. deciduous) (Czwienczek, 2012; Galmán et al., 2018). For example, global analysis of elevational gradients revealed decrease in herbivory with an increase in elevation in woody species, but not in non‐woody species; moreover, among woody species, this elevational decrease in herbivory was found in deciduous species, but not in evergreen species (Galmán et al., 2018). Consequently, herbivory on a given plant species may not be representative of the pattern observed at the level of the entire plant community (Zvereva et al., 2020). Although the hypothesis regarding the decrease in herbivory with increasing latitude and elevation was originally formulated as a community‐level hypothesis (Anstett et al., 2016; Coley & Barone, 1996), it has rarely been tested for elevational gradients with data on herbivory across the entire plant community.

The identification of the general (community‐wide) elevational pattern in herbivory requires simultaneous analysis of herbivory on all plant species, or at least on those dominating the community (Rheubottom et al., 2019; Zvereva et al., 2020). This task, which is challenging in the tropics, is relatively easy to perform at high latitudes. For example, in the mountains of the Kola Peninsula, tree and tall shrub species growing in the forest can also be found in the alpine tundra, and the field layer vegetation from forests to alpine tundra is dominated by the same species of dwarf shrubs (Alekseenko et al., 2017; Mishkin, 1953). Therefore, herbivory can be assessed on the same plant species across the entire elevation gradient, thereby avoiding the confounding effect of difference in plant species composition between different elevations.

Our goal was to test the hypothesis on the decrease in herbivory with increasing elevation in Arctic mountains. We asked whether (i) leaf herbivory demonstrates an overall decrease across the most abundant plant species; (ii) elevational changes in herbivory differ between plant species; (iii) variation in elevational patterns in herbivory between plant species is explained by plant life history traits (e.g., leaf longevity) and growth forms (dwarf or tall woody plants); and (iv) herbivory increases with an experimental increase in air temperature. We answered these questions by measuring leaf area losses to herbivores in naturally growing plants, some of which were enclosed within open‐top chambers.

## 2 MATERIALS AND METHODS

### 2.1 Study area

**FIGURE 1:**
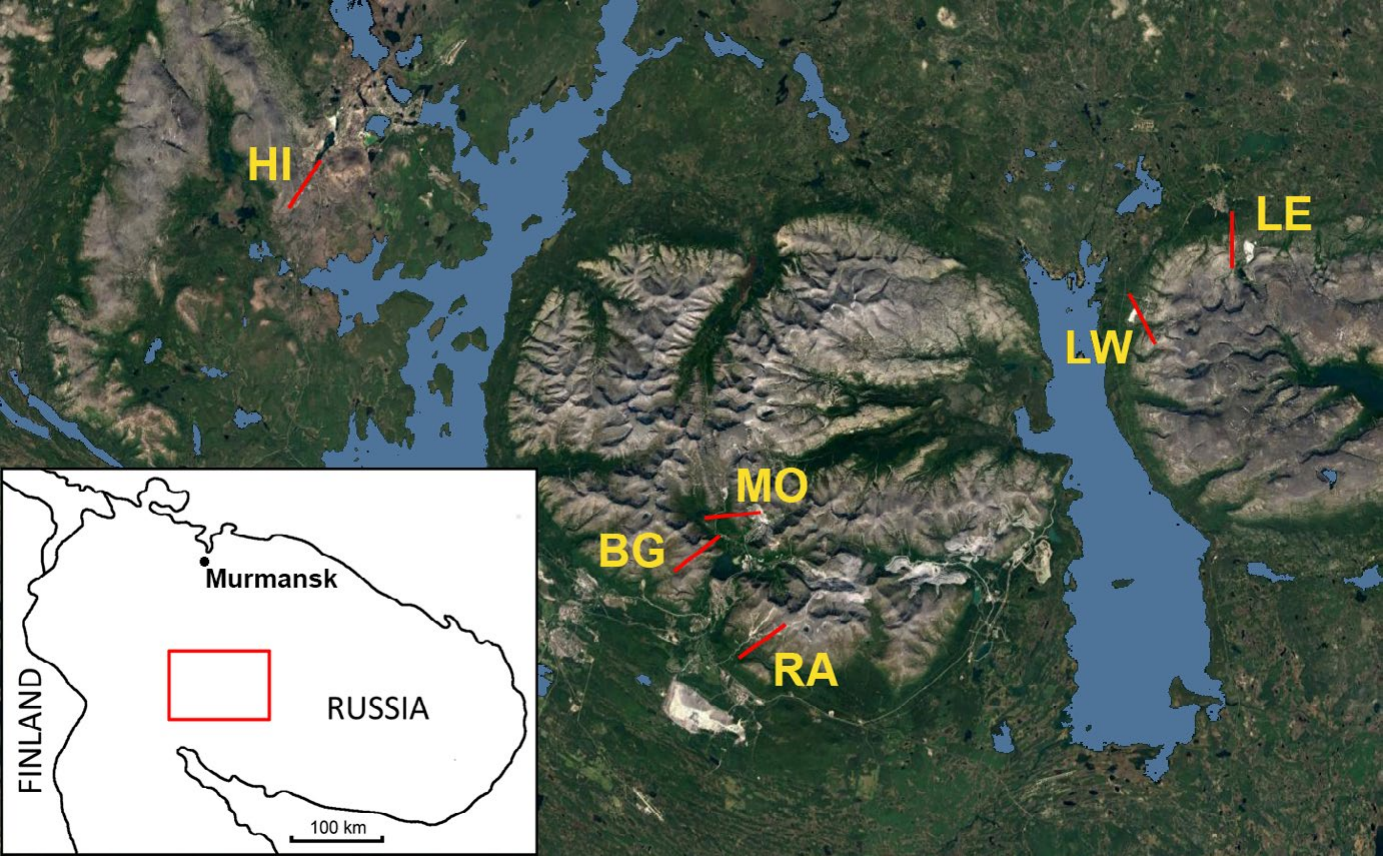
Location of the six elevational gradients. For plot coordinates and elevations, consult Table S1. Insert: position of the study area within the Kola Peninsula

The study was conducted in the central part of the Kola Peninsula in northwestern Russia (Figure 1). This region, which includes three mountain ranges (the Monche‐ and Chuna‐tundras and the Khibiny and Lovozero Mountains), provides a unique opportunity to replicate elevational gradients in mountains with different geochemistry and on slopes of different orientation. The lowest parts of the study region are located at the shores of Lake Imandra, the surface of which lies 127 m above sea level. The studied mountain ranges have altitudes that reach 1191 m (Khibiny), 1120 m (Lovozero), and 761 m (Monche‐tundra).

**FIGURE 2:**
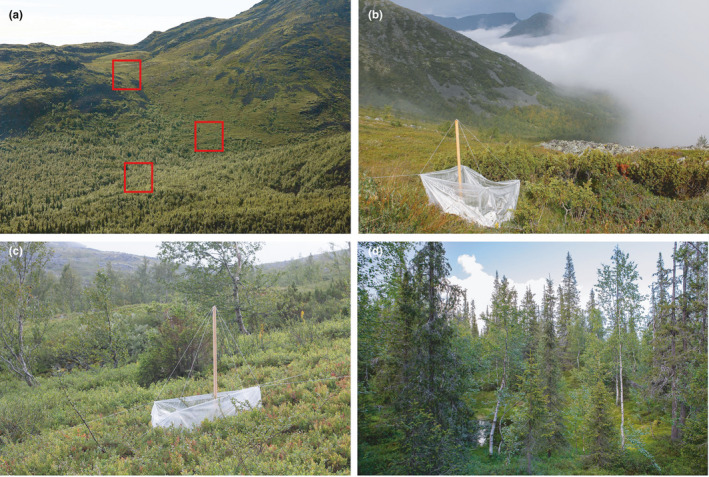
Landscapes of the study region and open‐top chambers: (a) the BG gradient on the northeastern slope of Mount Vudjavrchorr (red frames indicate approximate positions of study sites); (b) open‐top chamber in alpine tundra at the BG gradient; (c) open‐top chamber in a subalpine woodland at the HI gradient on the northeastern slope of the Monche‐tundra; (d) coniferous forest at the LW gradient on the western slope of Mount Alluaiv

The forests of the study region (Figure 2a,d) are dominated by Scots pine (*Pinus sylvestris* L.) and Norway spruce (*Picea abies* (L.) Karst.). The elevational tree line, located 350–450 m above sea level (Figure 2a,c), is formed by sparse, low‐stature woodlands of mountain birch (*Betula pubescens* var. *pumila* (L.) Govaerts). Above the tree line, in the mountain tundra (Figure 2b), solitary stunted (but not creeping) individuals of Scots pine, Norway spruce, and mountain birch, growing 50–200 m apart, occur up to 500–650 m above sea level, depending on the locality. Field‐layer vegetation in all sites consists primarily of crowberry, *Empetrum nigrum* ssp. *hermaphroditum* (Hagerup) Böcher, bilberry, *Vaccinium myrtillus* L., and lingonberry, *V. vitis‐idaea* L.

The summer in the study region is cool and short, lasting from 2 to 3 months. In the forested (lowland) sites, the mean temperature in January ranges from −11 to −14°C and in July from +11 to +14°C, with an annual precipitation from 450 to 560 mm. The frost‐free period varies from 50 to 100 days, but snow may occasionally occur even in mid‐summer.

### 2.2 Study sites and study plants

The study was replicated at four hierarchical levels: three mountain ranges, six gradients, three elevations within each gradient corresponding to three vegetation types, and 10 plant species. We selected our uppermost sites in alpine tundra (high elevation hereafter), at the upper distribution limit of our study trees (Scots pine, Norway spruce, and mountain birch). Our intermediate sites (mid‐elevation, hereafter) were located in subalpine birch woodland. The lowest sites (low elevation, hereafter) were chosen in closed canopy coniferous forests at the foot of the respective mountains. The difference in elevation between the tundra and forest sites ranged from 170 to 325 m (Table S1).

We measured herbivory in ten plant species: five trees and tall shrubs (evergreen *P*. *abies*, *P. sylvestris*, and *Juniperus communis* L.; deciduous *B*. *pubescens* and *Salix phylicifolia* L.) and five low shrubs (evergreen *V. vitis‐idaea* and deciduous *V*. *myrtillus*, *V*. *uliginosum* L., *S*. *glauca* L., and *B*. *nana* L.). Four of these species (*B*. *pubescens* and three *Vaccinium* species) were present in all sites, whereas other species each occurred on 5‒17 plots (Table S2). Based on the density of trees and shrubs and on the cover of the field‐layer vegetation (unpublished data), we estimate that, on average, our study plants jointly contributed over 80% to the foliar biomass of the explored plant communities and that the foliar biomass in the mountain tundra sites was 1–5% of that observed in the forest sites.

### 2.3 Experimental warming

In the early summer of 2012, at each of six study sites (located in HI and BG gradients), we selected five pairs of study plots (1 × 1 m size) for experimental treatments. One randomly selected plot from each pair was enclosed in a passive open‐top greenhouse made of polyethylene, with 50 cm walls surrounding the study plots (Figure 2b,c); the second plot served as control. The chambers in the BG sites were established on June 25, 2012, and those in the HI sites on June 27, 2012.

### 2.4 Sampling and processing

The branches of the study plants were collected at the beginning of autumn (August 13‒17, 2012, August 8‒12, 2013, and August 8‒15, 2014), when most insect herbivores had completed their development. We haphazardly (on a first found, first sampled basis) selected five mature individuals (or patches) of each of our study species present at a site while standing at a distance of 5‒10 m away to avoid unconscious selection bias. Each selected plant/patch was located at least 10 m apart from others of its species, and each collected branch (or group of stems) generally contained 100–200 leaves. In 2012, at sites in the BG and HI gradients, we also collected samples of *S*. *glauca*, *B*. *nana*, *V*. *uliginosum*, *V. myrtillus*, and *V. vitis‐idaea* from the study plots enclosed in open‐top chambers.

In the laboratory, each leaf was carefully examined for the presence of damage imposed by chewing insect herbivores (both miners and defoliators). In conifers, we searched for traces of insect feeding in 50 or 100 current‐year needles, starting from the tip of the branch, and we counted needles that were missing from the shoot. As in the previous study (Zvereva et al., 2020), we attributed the loss of entire needles on current‐year shoots to herbivory because undamaged needles of conifers in our study region persist for several years (Kozlov et al., 2009). Mechanical damage to leaves and needles rarely occurs in our study region, and it can be easily distinguished from herbivory. Both our observations and published data on the plant‐feeding organisms of Northern Europe indicate that all the types of damage recorded in the course of our study were imposed by insects.

Following a widely used methodology (Alliende, 1989; Kozlov et al., 2015), each leaf/needle (leaf hereafter) was assigned to one of the damage classes according to the percentage of the leaf area that was consumed or otherwise damaged by insects: 0 (intact leaves), 0.01%–1%, 1%–5%, 5%–25%, 25%–50%, 50%–75%, 75%–99%, and 100%. The last class included petioles of fully consumed leaves and missing needles. The foliage lost to insects (i.e., the leaf herbivory level) was calculated as follows: the numbers of leaves in each damage class were multiplied by the respective median values of the damaged leaf area (i.e., 0 for intact leaves, 0.5% for the damage class 0.01%–1%, 3% for the damage class 1%–5%, etc.); the obtained values were then summed for all damage classes and divided by the total number of leaves (including undamaged ones) in a sample.

In 2012, we also measured the specific leaf area (SLA) from all seven species of leaf‐bearing plants. For this purpose, we made 2‒5 discs 4.5 or 8 mm in diameter (depending on leaf size) from five haphazardly selected leaves, dried the discs for 24 h at 105°C and then weighed them to the nearest 0.1 mg. SLA was then calculated as total area of the discs (mm^2^) divided by dry weight (mg).

### 2.5 Data analysis

We modelled the proportion of insect damage in leaves using a GLMM approach (SAS GLIMMIX procedure; SAS Institute, 2009) with a beta error distribution and a logit link function. In this model, the elevation level, plant species, study year, and their interactions were considered fixed effects, whereas the gradient was treated as a random effect. The same model, with the addition of one more fixed effect (treatment), was used to test for the effects of experimental warming on herbivory. The effects of leaf longevity (evergreen vs. deciduous) and plant height (trees and tall shrubs vs. low shrubs) were tested by adding these fixed effects (one by one) to the model. In these models, plant species were nested within the respective groups (i.e., leaf longevity and plant height). Similar models with Gaussian distribution were employed to explore the variation in SLA. We preferred the model with elevational level as the fixed effect to the model with the absolute value of elevation as a covariate because effect of elevation on herbivory in the latter model appeared non‐significant (*F*_1,18.8_ = 0.10, *p* = .76). We adjusted the standard errors and denominator degrees of freedom following Kenward and Roger (2009) and evaluated the significance of a random factor by testing the likelihood ratio against the Chi‐squared distribution (Littell et al., 2006).

## 3 RESULTS

### 3.1 Elevational changes in herbivory and SLA

**FIGURE 3:**
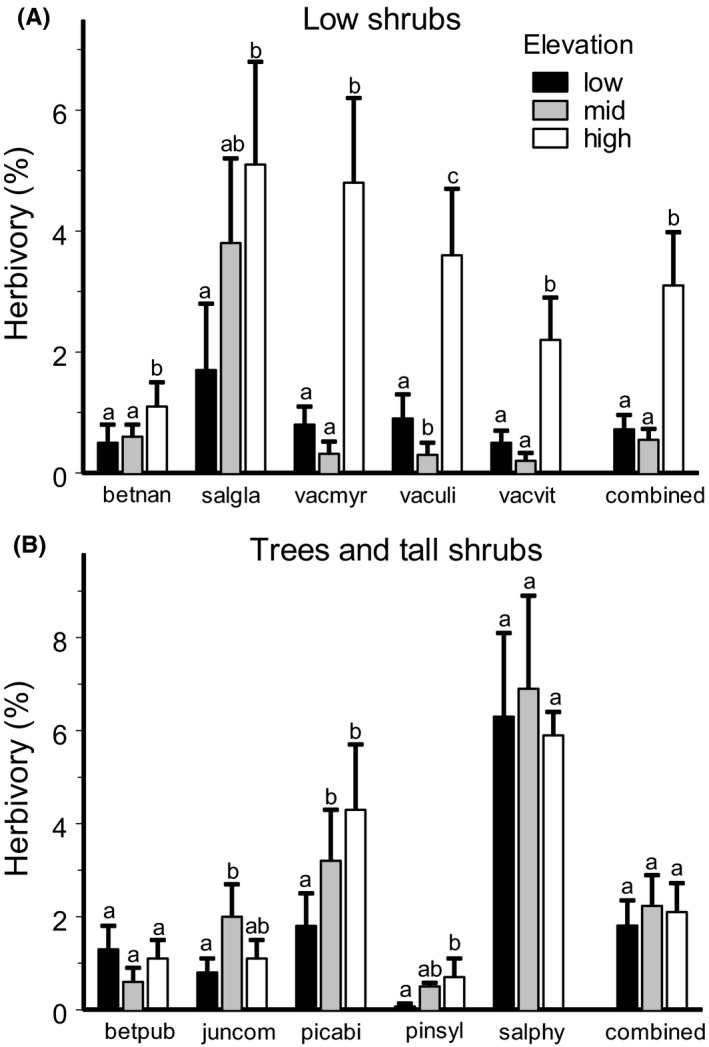
Insect herbivory at forest (low), subalpine woodland (mid), and alpine tundra (high) sites for low shrubs (A) and trees and tall shrubs (B); by species and combined within each group. The estimated marginal means (+SE) are based on data collected from six elevational gradients (consult Figure 1) from 2012 to 2014. Species: betnan, *Betula nana*; salgla, *Salix glauca*; vacmyr, *Vaccinium myrtillus*; vaculi, *V*. *uliginosum*; vacvit, *V. vitis‐idaea*; betpub, *B*. *pubescens*; juncom, *Juniperus communis*; picabi, *Picea abies*; pinsyl, *Pinus sylvestris*; and salphy, *S*. *phylicifolia*. Bars with different letters indicate significant (*p* < .05) differences between elevations (*t* test from SAS GLIMMIX procedure)

**FIGURE 4:**
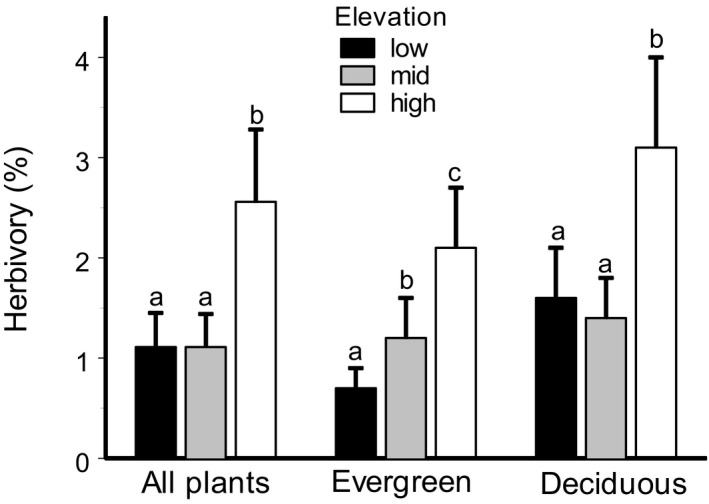
Insect herbivory at forest (low), subalpine woodland (mid), and alpine tundra (high) sites for 10 dominant plant species, combined and separately, for evergreen (4 species) and deciduous (6 species) plants. The estimated marginal means (+SE) are based on data collected from six elevation gradients (consult Figure 1) from 2012 to 2014. Bars with different letters indicate significant (*p* < .05) differences between elevations (*t* test from SAS GLIMMIX procedure)

The leaf area lost to insects varied significantly among elevation levels, study years, plant species, and gradients (Table 1). The elevational changes in herbivory differed between species (Table 1, Figure 3); however, on average, herbivory at high elevations was 2.2‐fold greater compared with either mid or low elevations (Figure 4). This pattern was similar in evergreen and deciduous species (leaf longevity × elevation level interaction: *F*_2,1890_ = 2.91, *p* = .055), but an increase in herbivory was already observed in evergreen species at mid‐elevation sites and further increased to high elevations, whereas the deciduous species showed similar herbivory levels at low‐elevation and mid‐elevation sites (Figure 4).

**TABLE 1:** Effects of elevation level (low, mid, and high), study year, and plant species on the insect herbivory and on the specific leaf area (SAS GLIMMIX procedure, type 3 tests)

Effect type	Explanatory variable	Herbivory		Specific leaf area	
		Test statistics	*p* value	Test statistics	*p* value
Fixed	Elevation level	*F*_2,1858_ = 28.72	<.0001	*F*_2,439.5_ = 24.24	<.0001
	Year	*F*_2,1858_ = 7.67	.0005	–	–
	Species	*F*_9,1860_ = 33.22	<.0001	*F*_6,439.8_ = 112.2	<.0001
	Year × Elevation level	*F*_4,1858_ = 1.93	.1030	–	–
	Elevation level × Species	*F*_18,1858_ = 5.39	<.0001	*F*_12,439.2_ = 1.05	.4024
	Year × Species	*F*_18,1858_ = 7.38	<.0001	–	–
Random	Gradient	$\chi_{1}^{2}$ = 239.78	<.0001	$\chi_{1}^{2}$ = 92.43	<.0001

Changes in herbivory with elevation differed considerably between low shrubs and taller plants (plant height × elevation level interaction: *F*_2,1890_ = 32.6, *p* < .0001). Herbivory on low shrub species was fourfold greater at high elevation sites than at lower elevations (Figure 3a). By contrast, herbivory on trees and tall shrubs generally did not change with elevation (Figure 3b).

**FIGURE 5:**
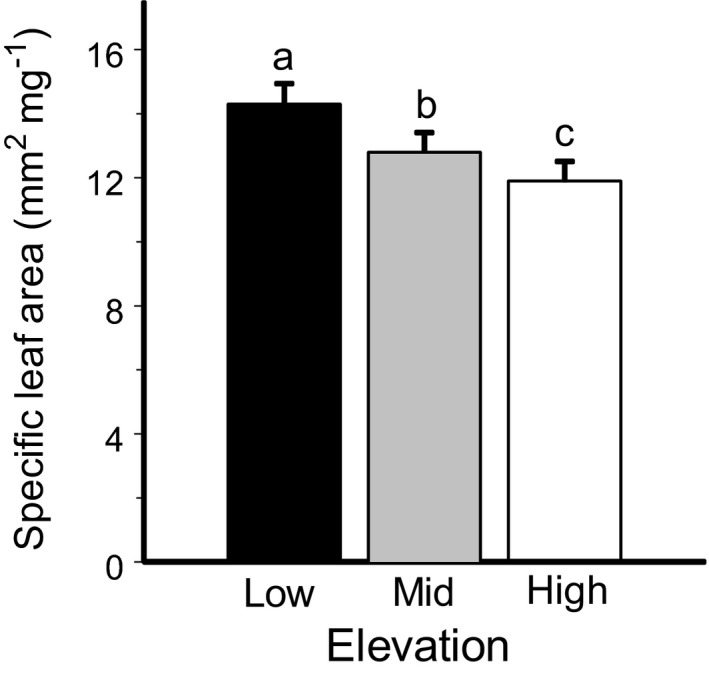
Specific leaf area of seven leaf‐bearing species growing in forest (low), subalpine woodland (mid), and alpine tundra (high) sites. The estimated marginal means (+SE) are based on data collected from six gradients (consult Figure 1) in 2012. Bars with different letters indicate significant (*p* < .05) differences between elevations (*t* test from SAS GLIMMIX procedure)

The SLA of leaf‐bearing plants significantly decreased from low to mid elevations and further decreased from mid to high elevations (Table 1, Figure 5). At high elevations, the SLA comprised 83% of the values observed at low elevations. This trend was similar in both low and tall species (plant height × elevation level interaction: *F*_2,449.0_ = 0.79, *p* = .46).

### 3.2 Effects of chambers on herbivory and SLA

**FIGURE 6:**
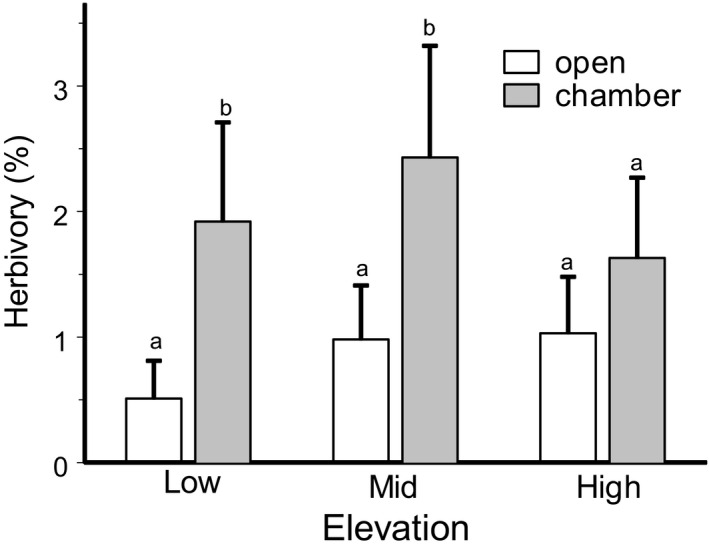
Insect herbivory in passive open‐top chambers and in open patches in forest (low), subalpine woodland (mid), and alpine tundra (high) sites. The estimated marginal means (+SE) are based on data collected from five species of low shrubs (consult Figure 3) and two elevational gradients (HI and BG, consult Figure 1) in 2012. Within each elevation, bars labeled with different letters indicate significant (*p* < .05) differences between plants enclosed in chambers and control (open) plants (*t* test from SAS GLIMMIX procedure)

Plants enclosed in open‐top chambers suffered significantly higher herbivory than was observed in control plants (*F*_1,194.0_ = 13.12, *p* = .0004), but the magnitude of this effect changed with elevation (Table S3). The chamber effect on herbivory was highest (3.2‐fold) at low elevations, decreased to 2.5‐fold in mid‐elevations, and became non‐significant (1.5‐fold) at high‐elevation sites (Figure 6). Plant inclusion in the chamber did not affect the SLA (Table S3).

## 4 DISCUSSION

The elevational increase in herbivory detected in our study was strong and robust, despite significant variations in the elevational changes between the six gradients and 10 woody plant species. This pattern, that is, an increase in herbivory with elevation, has been reported in 22% of elevational gradients located in both tropical and temperate mountains (reviewed by Moreira et al., 2018). However, whether the previously detected elevational increase in herbivory should be attributed to the peculiar responses of studied plant species or to the specificity of elevational gradients remains unclear.

Variations in changes in insect herbivory among plant species within a single elevational gradient have been reported in several earlier studies (Czwienczek, 2012; Scheidel & Bruelheide, 2001). We also detected a significant among‐species variation in elevational changes; nevertheless, we observed an increase in herbivory with elevation in 8 of 10 plant species, whereas herbivory in the two remaining species did not change with elevation (Figure 3). Our study included all plant species commonly found in our sites and which jointly comprise over 80% of the foliar biomass in the local plant communities. Therefore, the detected increase in herbivory from forest to alpine tundra (Figure 4) likely reflects a community‐wide pattern. This gives special importance to our findings because the elevational changes in herbivory reported in previous case studies (reviewed by Moreira et al., 2018) were based on one or a few plant species, so they may not reflect the elevational changes in the role of herbivory in mountain ecosystems.

One factor suggested to explain spatial patterns in herbivory is variation in host plant quality, particularly in plant defenses (reviewed by Carmona et al., 2020). The elevational changes in anti‐herbivory defenses differ among plant species (Moreira et al., 2018), and the previous studies documented almost all possible associations between herbivory and plant physical and chemical defenses along elevational gradients. These included a simultaneous decrease in both herbivory and plant defense (Pellissier et al., 2014), a decrease in herbivory accompanied by an increase in plant defenses (Rasmann et al., 2014) and, finally, simultaneous increases in both insect herbivory and anti‐herbivore defense from low to high elevations (Abdala‐Roberts et al., 2016; Galmán et al., 2019). Our results are in line with the last pattern because the increase in herbivory with elevation occurs despite the decrease in SLA. SLA, which reflects leaf physical properties, is often interpreted as physical antiherbivore defense (Barton & Koricheva, 2010; Galmán et al., 2019; Kergunteuil et al., 2018; Wilson et al., 1999). Furthermore, SLA correlates with other leaf traits (e.g., with foliar nitrogen) that influence plant quality for herbivores (Reich et al., 1999), and therefore the decrease in SLA with elevation observed in our study (and in several previous studies: Descombres et al., 2020; Galmán et al., 2019; Garibaldi et al., 2011) likely indicates the decrease in plant quality. However, herbivory in plants enclosed in the open‐top chambers increased despite the absence of changes in SLA, thereby casting doubt on a causal link between SLA and herbivory in our study. Although we cannot exclude the elevational decrease in plant chemical defenses, our data support the conclusion (Abdala‐Roberts et al., 2016; Czwienczek, 2012; Galmán et al., 2019) that elevational changes in herbivory are unlikely driven by elevational changes in bottom‐up factors.

Top‐down factors can also shape environmental gradients in herbivory (Björkman et al., 2011; Roslin et al., 2017). Parasitism and predation on herbivorous insects and the density of predatory groups frequently decrease with elevation (Bowden & Buddle, 2010; Hodkinson, 2005; Pepi et al., 2017). However, other studies did not find any elevational trends in bird predation on arthropods (Schwenk et al., 2010; Zehnder et al., 2010). In particular, a bird exclusion experiment previously conducted in three of our six gradients showed similar effects of bird predation on insect herbivory in alpine tundra and in lowland forests (Zverev et al., 2020). Moreover, the density of invertebrate predators in the Khibiny Mountains was higher in the alpine tundra than in the forests (Zenkova et al., 2011). Therefore, we conclude that the increase in herbivory with elevation in our study region is unlikely shaped by the elevational changes in top‐down factors.

The most important environmental factors that affect herbivory (solar radiation, temperature, and soil moisture) all change with elevation in a concerted manner (Lookingbill & Urban, 2005). Among these, the temperature gradient is most frequently used to explain changes in biota in elevational studies (Körner, 2007). Manipulating the temperature by passive climate chambers allowed us to disentangle the effects of air temperature and other environmental factors on insect herbivory.

Our chambers were similar in size to the chambers used in the International Tundra Experiment (Marion et al., 1997), which were found to increase the mean air temperature by ca. 1.5°C during the growing season (Klanderud & Totland, 2005; Sandvik & Eide, 2009). The higher levels of insect herbivory in our chambers relative to the control (open) microsites are consistent with the outcomes of open‐top chamber experiments conducted at alpine sites in Norway (Birkemoe et al., 2016), and they confirm the importance of temperature in shaping plant damage by insects in our study sites. The positive association between herbivory and temperature is consistent with the elevational decrease in herbivory demonstrated in many case studies (reviewed by Moreira et al., 2018) and identified as the general regularity for woody plants in the global analysis (Galmán et al., 2018); however, it opposes the elevational increase in herbivory detected in our study. This contradiction could be resolved if the ambient temperatures in our gradients do not decrease with elevation.

Our elevational gradients are relatively short (an average difference in elevation between the alpine and forest sites is ca. 200 m); therefore, the temperature decrease from the lowest to the highest sites expected due to lapse rate is low compared with elevational studies conducted in tropical and temperate zones (average difference in elevation 1340 m, as calculated from Moreira et al., 2018). Based on a generally accepted lapse rate of −0.55°C/100 m (Körner, 2007), we estimate that the ambient air temperature should drop by ca. 1°C between our forest and alpine tundra sites. However, the air temperatures measured in one of our gradients (BG) did not show a steady decrease from forest to subalpine and then to alpine sites (mean June temperatures 5.8, 5.3, and 5.6°C: Zenkova et al., 2011). This lack of an elevational decrease in temperatures, and even an increase in temperature with an increase in elevation (i.e., positive lapse rate), was also observed on several other mountains, mainly those located at high latitudes (Graae et al., 2012; Pepin et al., 2009; Wundram et al., 2010).

The lapse rate is generally calculated from temperatures measured in shadows 2 m above the ground. However, these temperatures are of lesser importance in alpine and Polar communities compared to the microclimate at the soil surface (Körner, 2007). Insolation of soil and plants in open alpine habitats may lead to considerable increases in summer temperatures near the soil surface; for example, temperatures of alpine cushion plants under full solar irradiance can reach 25‒30°C at air temperatures around 10°C (Körner, 2007). By contrast, solar radiation in forests is intercepted by the tree canopies, resulting in lower soil temperatures. For example, summer soil temperatures in the Alps are higher in alpine grasslands than in lowland forests (Körner, 1998). Similarly, the summer soil surface temperatures were higher in the alpine tundra in one of our gradients in the Khibiny Mountains than in the subalpine birch woodland (Shtambovskaja & Zenkova, 2018). In other localities of the Khibiny Mountains, the mean soil surface temperature was 6°C higher in the alpine tundra than at the upper border of the continuous forest, located 500 m below the tundra site (Kryuchkov, 1958). The critical importance of habitat openness for elevational changes in herbivory observed in our study is supported by sharp increase in herbivory in treeless (alpine) habitats.

The importance of near‐surface temperatures for insect herbivores in our gradients is strongly supported by the elevational increase in herbivory observed in low‐stature plants (Figure 3a), whose leaves (and the insects feeding on these leaves) experience the near‐surface temperatures. At the same time, herbivory on tall plants, whose leaves are located further away from the soil surface and are therefore exposed to colder air, generally did not change with elevation (Figure 3b). We suggest that a shallower or even an inverse elevational gradient in near‐surface temperatures compared with air temperatures 2 m above the ground results from the higher openness of the alpine sites in the majority of elevational gradients studied so far. This difference between near‐surface and air temperatures may explain why previous global analysis (Galmán et al., 2018) discovered elevational changes in herbivory in woody plants (mostly tall trees) but not in herbaceous (mostly low‐stature) plants.

Two factors affect the elevational gradients in temperatures at the soil surface: the temperature lapse (driving the overall temperature decrease with elevation) and the elevational changes in vegetation, expressed as increasing openness leading to soil warming due to high irradiation. In the polar mountains, open alpine zone starts at relatively low elevations (Körner, 1998), where temperature decreases due to lapse rate are small. Therefore, an increase in near‐surface temperatures in alpine tundra habitats in the polar mountains may appear greater than temperature decrease due to lapse rate, thus resulting in the inverse gradients in temperatures at the soil surface. We suggest that the increase in herbivory from forest to alpine tundra sites observed in our study may be related to these inverse gradients in temperatures at the soil surface. Verification of this hypothesis requires further studies of the elevational patterns in herbivory at high latitudes.

## 5 CONCLUSION

Our data from six elevational gradients, in combination with other studies addressing the effects of elevation on plant‐feeding insects and insect herbivory at high latitudes (Kristensen et al., 2020; Mjaaseth et al., 2005; Pepi et al., 2017; Ruohomäki et al., 1997), indicate that an increase in herbivory with elevation is more frequent in Arctic mountains than in mountains at lower latitudes (Moreira et al., 2018). This pattern, which contrasts with both theoretical predictions and the results of the analysis of elevational changes in herbivory in tropical and temperate mountains (Galmán et al., 2018), likely emerges due to an inverse elevational gradient in temperatures at the soil surface between closed‐canopy forests and open alpine habitats.

## CONFLICT OF INTEREST

Authors do not have any conflict of interest.

## AUTHOR CONTRIBUTION

**Elena L. Zvereva:** Conceptualization (lead); Methodology (equal); Writing – original draft (lead). **Vitali Zverev:** Investigation (equal); Methodology (equal); Resources (equal); Visualization (equal); Writing – review & editing (equal). **Mikhail V. Kozlov:** Conceptualization (equal); Data curation (equal); Formal analysis (equal); Funding acquisition (equal); Methodology (equal); Writing – review & editing (equal).

## Supporting information

Supplementary Material

## Data Availability

Data available from the Dryad Digital Repository: https://doi.org/10.5061/dryad.7m0cfxpw6.
