# Supplementary material for: Insect herbivory increases from forest to alpine tundra in Arctic mountains
Source: Ecol Evol. 2022 Jan 24;12(1):e8537. doi: 10.1002/ece3.8537 (PMC8796911; doi:10.1002/ece3.8537)
Supplement: Supplementary file 1 — Supplementary Material [file ECE3-12-e8537-s001.doc]

**Insect herbivory increases from forest to alpine tundra in Arctic mountains**

Elena L. Zvereva*, Vitali Zverev and Mikhail V. Kozlov

Department of Biology, University of Turku, 20014 Turku, Finland

*elezve@utu.fi

**Supporting information**

**Table S1. Characteristics of study sites.**

| Mountain ridge | Gradient name | Slope orientation | Habitat | Elevation level | Altitude, m a.s.l. | Latitude,  N | Longitude, E |
| --- | --- | --- | --- | --- | --- | --- | --- |
| Lovozero | LC | East | Alpine | High | 465 | 67°53´29˝ | 34°34´54˝ |
|  |  |  | Subalpine | Mid | 405 | 67°54´02˝ | 34°36´02˝ |
|  |  |  | Forest | Low | 295 | 67°55´58˝ | 34°34´49˝ |
|  | LW | West | Alpine | High | 475 | 67°51´59˝ | 34°26´15˝ |
|  |  |  | Subalpine | Mid | 425 | 67°51´56˝ | 34°25´43˝ |
|  |  |  | Forest | Low | 305 | 67°53´14˝ | 34°25´17˝ |
| Monche-tundra | HI | North-East | Alpine | High | 430 | 67°51´42˝ | 32°44´52˝ |
|  |  |  | Subalpine | Mid | 360 | 67°51´59˝ | 32°45´43˝ |
|  |  |  | Forest | Low | 190 | 67°53´04˝ | 32°47´03˝ |
| Khibiny | BG | North-East | Alpine | High | 530 | 67°38´37˝ | 33°36´11˝ |
|  |  |  | Subalpine | Mid | 445 | 67°38´47˝ | 33°39´08˝ |
|  |  |  | Forest | Low | 355 | 67°38´58˝ | 33°39´11˝ |
|  | MO | West | Alpine | High | 555 | 67°40´06˝ | 33°40´18˝ |
|  |  |  | Subalpine | Mid | 425 | 67°40´01˝ | 33°39´25˝ |
|  |  |  | Forest | Low | 365 | 67°39´23˝ | 33°39´14˝ |
|  | RA | South-West | Alpine | High | 630 | 67°35´16˝ | 33°45´47˝ |
|  |  |  | Subalpine | Mid | 470 | 67°34´54˝ | 33°43´53˝ |
|  |  |  | Forest | Low | 305 | 67°34´10˝ | 33°41´26˝ |

**Table S2.** The occurrence of plant species in study sites (H, high elevation: alpine tundra; M, mid-elevation: subalpine woodland; L, low elevation: coniferous forest) and gradients. For gradient positions, consult Fig. 1 and Table S1.

| Вид | LC | | | LW | | | HI | | | BG | | | MO | | | RA | | |
| --- | --- | --- | --- | --- | --- | --- | --- | --- | --- | --- | --- | --- | --- | --- | --- | --- | --- | --- |
|  | H | M | L | H | M | L | H | M | L | H | M | L | H | M | L | H | M | L |
| *Picea abies* |  |  |  |  |  |  | + | + | + | + | + | + | + | + | + | + | + | + |
| *Pinus sylvestris* |  |  |  | + |  | + | + | + | + |  |  |  |  |  |  |  |  |  |
| *Juniperus communis* | + | + | + | + |  | + | + | + | + | + | + | + | + | + | + | + | + | + |
| *Betula pubescens* | + | + | + | + | + | + | + | + | + | + | + | + | + | + | + | + | + | + |
| *Salix glauca* |  |  |  |  |  |  | + | + | + | + | + |  | + |  | + | + | + |  |
| *Salix phylicifolia* | + | + | + | + | + | + |  |  |  |  |  |  |  |  |  | + | + | + |
| *Betula nana* | + | + |  | + | + | + | + | + | + | + | + |  | + | + |  | + | + |  |
| *Vaccinium vitis-idaea* | + | + | + | + | + | + | + | + | + | + | + | + | + | + | + | + | + | + |
| *Vaccinium uliginosum* | + | + | + | + | + | + | + | + | + | + | + | + | + | + | + | + | + | + |
| *Vaccinium myrtillus* | + | + | + | + | + | + | + | + | + | + | + | + | + | + | + | + | + | + |

**Table S3.** Effect of open-top chambers on the insect herbivory and the specific leaf area (SAS GLIMMIX procedure, type 3 tests).

| Effect type | Explanatory variable | Herbivory | | Specific leaf area | |
| --- | --- | --- | --- | --- | --- |
|  |  | Test statistics | *P* value | Test statistics | *P* value |
| Fixed | Elevation | *F*_2, 194.3_ = 0.69 | 0.50 | *F*_2, 195.1_ = 6.01 | 0.0029 |
|  | Species | *F*_4, 194.1_ = 6.28 | <0.0001 | *F*_1, 195.0_ = 76.46 | <0.0001 |
|  | Treatment | *F*_1, 194.0_ = 13.12 | 0.0004 | *F*_1, 195.0_ = 0.11 | 0.74 |
|  | Elevation × Species | *F*_8, 194.1_ = 2.25 | 0.0255 | *F*_8, 195.0_ = 1.64 | 0.12 |
|  | Elevation × Treatment | *F*_2, 194.0_ = 1.33 | 0.27 | *F*_2, 195.0_ = 0.49 | 0.62 |
|  | Species × Treatment | *F*_4, 194.0_ = 0.65 | 0.63 | *F*_4, 195.0_ = 0.41 | 0.80 |
| Random | Gradient | *χ*^2^_1_ = 4.79 | 0.0143 | *χ*^2^_1_ = 69.92 | <0.0001 |
